# Antimicrobial, Antioxidant, and Anticancer Activities of Biosynthesized Silver Nanoparticles Using Marine Algae *Ecklonia cava*

**DOI:** 10.3390/nano6120235

**Published:** 2016-12-06

**Authors:** Jayachandran Venkatesan, Se-Kwon Kim, Min Suk Shim

**Affiliations:** 1 Division of Bioengineering, Incheon National University, Incheon 406-772, Korea; venkatjchem@gmail.com; 2 Marine Bioprocess Research Center, Pukyong National University, Busan 608-737, Korea; sknkim@pknu.ac.kr

**Keywords:** anticancer, antimicrobial, antioxidant, biosynthesis, *Ecklonia cava*, nanoparticle

## Abstract

Green synthesis of silver nanoparticles (AgNPs) has gained great interest as a simple and eco-friendly alternative to conventional chemical methods. In this study, AgNPs were synthesized by using extracts of marine algae *Ecklonia cava* as reducing and capping agents. The formation of AgNPs using aqueous extract of *Ecklonia cava* was confirmed visually by color change and their surface plasmon resonance peak at 418 nm, measured by UV-visible spectroscopy. The size, shape, and morphology of the biosynthesized AgNPs were observed by transmission electron microscopy and dynamic light scattering analysis. The biosynthesized AgNPs were nearly spherical in shape with an average size around 43 nm. Fourier transform-infrared spectroscopy (FTIR) analysis confirmed the presence of phenolic compounds in the aqueous extract of *Ecklonia cava* as reducing and capping agents. X-ray diffraction (XRD) analysis was also carried out to demonstrate the crystalline nature of the biosynthesized AgNPs. Antimicrobial results determined by an agar well diffusion assay demonstrated a significant antibacterial activity of the AgNPs against *Escherichia coli* and *Staphylococcus aureus*. Antioxidant results determined by 1,1-diphenyl-2-picrylhydrazyl (DPPH) scavenging assay revealed an efficient antioxidant activity of the biosynthesized AgNPs. The biosynthesized AgNPs also exhibited a strong apoptotic anticancer activity against human cervical cancer cells. Our findings demonstrate that aqueous extract of *Ecklonia cava* is an effective reducing agent for green synthesis of AgNPs with efficient antimicrobial, antioxidant, and anticancer activities.

## 1. Introduction

In recent years, noble metal nanoparticles (NPs) have been intensively utilized for biomedical applications, such as diagnostics, drug delivery, and tissue engineering, due to their unique physicochemical and optoelectronic properties [1,2,3,4]. Among various noble metal nanoparticles, silver nanoparticles (AgNPs) have received great attention in a variety of applications, including nanoelectronic devices, sensors, imaging contrast agents, filters, and antimicrobial agents due to their good electrical conductivity, stability, optical property, and antimicrobial activity [5,6]. AgNPs have also extended their applications to cancer therapy. Several in vitro studies using AgNPs have demonstrated their potential as effective anticancer agents [7,8,9,10]. They have exhibited apoptosis-mediated, strong anticancer efficacies in a variety of cancer cells, including human cervical cancer [8], lung cancer [9], and breast cancer cells [10].

It has been well-documented that performance and applicability of AgNPs critically depend on their size, shape, composition, and surface chemistry [11,12,13]. Synthesis of noble metal NPs with controlled shape and size can be achieved through many different synthetic methods: evaporation-condensation, gamma irradiation, electron irradiation, microwave processing, microemulsion, sonochemical, electrochemical, photochemical, etc. [14,15,16,17]. One of the popular methods for the synthesis of defined noble metal NPs is chemical reduction [14]. Reduction of silver complexes in dilute solution with a proper reductant can lead to the formation of colloidal AgNPs [14]. Although this method offers significant advantages of simple equipment and convenient operation, it involves a use of hazardous chemicals and high temperature conditions, which are rather environmentally unfriendly and energetically inefficient [14,18].

To overcome the drawbacks of the chemical reduction method, biogenic synthesis that employs microorganisms and naturally occurring products has emerged as an environmentally friendly synthetic method (i.e., green chemistry) [17,19,20,21]. This method offers a facile and convenient entry to producing various noble metal NPs. Biogenic synthesis of noble metal NPs mainly relies on various extracts (e.g., nucleic acids, enzymes, proteins, peptides, vitamins, and polysaccharides) of microorganisms. For example, extracts of fungi, bacteria, and algae were extensively utilized to synthesize AgNPs, as shown in Table 1. 

**Figure 1:**
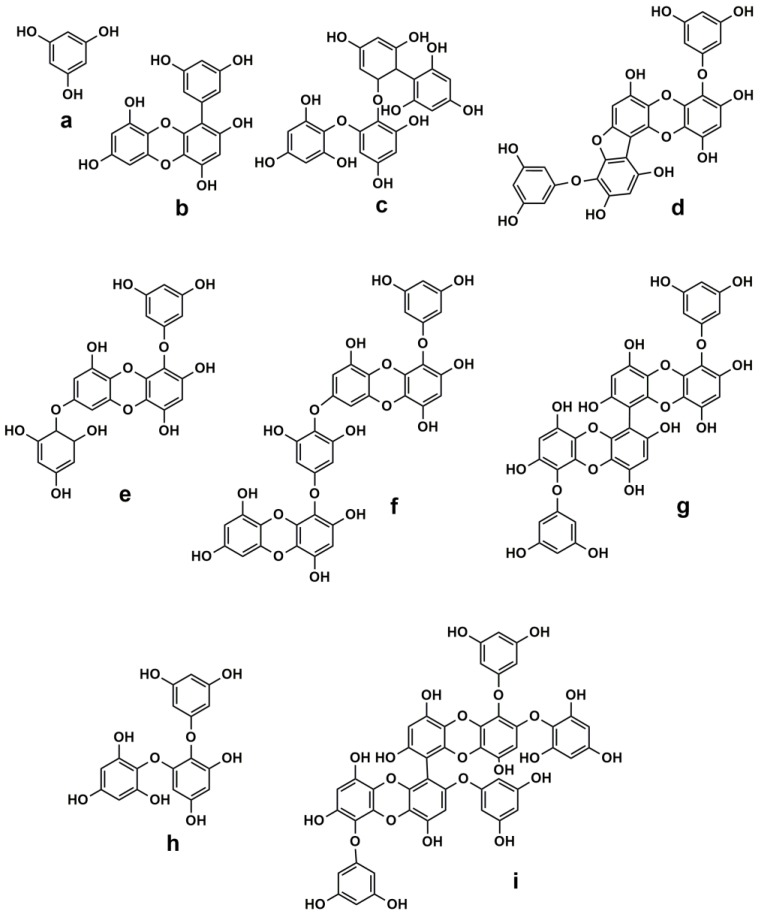
Chemical structures of: (**a**) phloroglucinol; (**b**) eckol; (**c**) fucodiphlorethol G; (**d**) phlorofucofuroeckol A; (**e**) 7-phloroeckol; (**f**) dieckol; (**g**) 6,6′-bieckol; (**h**) triphlorethol-A; and (**i**) 2,7′-phloroglucinol-6,6′-bieckol.

Seaweeds from the ocean are considered as “sea vegetables”, commonly used as food in Asia-Pacific areas such as Korea, China, and Japan. Seaweeds are often used to produce hydrocolloids such as alginate, agar, and carrageenan. *Ecklonia cava* is an edible brown alga, mainly present in Korea, Japan, and China. Previous studies reported that phlorotannins such as phloroglucinol are main components in *Ecklonia cava* [62,63,64,65]. These compounds in *Ecklonia cava* have proved to be responsible for bioactivities such as antioxidant [62,66], anticancer [67,68,69], and antimicrobial activities [70]. In addition, antioxidant compounds from *Ecklonia cava* extracts, including (a) phloroglucinol; (b) eckol; (c) fucodiphlorethol G; (d) phlorofucofuroeckol A; (e) 7-phloroeckol; (f) dieckol; (g) 6,6′-bieckol; (h) triphlorethol-A; and (i) 2,7′-phloroglucinol-6,6′-bieckol (Figure 1) [42,62,67,68,69,70,71], could act as effective reductants to synthesize noble metal NPs. 

In this study, AgNPs were synthesized via reduction of silver ions using aqueous extracts of *Ecklonia cava* as an alternative to conventional chemical reduction methods. To the best of our knowledge, use of *Ecklonia cava* extracts for the biosynthesis of AgNPs has not been attempted yet. We synthesize AgNPs and subsequently characterize their morphologies and compositions. We also investigate whether phloroglucinol and their derivatives obtained from *Ecklonia cava* contribute to the reduction of silver ions required for the formation of AgNPs. Antimicrobial, antioxidant, and anticancer activities of the biosynthesized AgNPs are also investigated. 

## 2. Results and Discussion

### 2.1. Biosynthesis of AgNPs and Characterization by UV-Vis Spectroscopy

**Figure 2:**
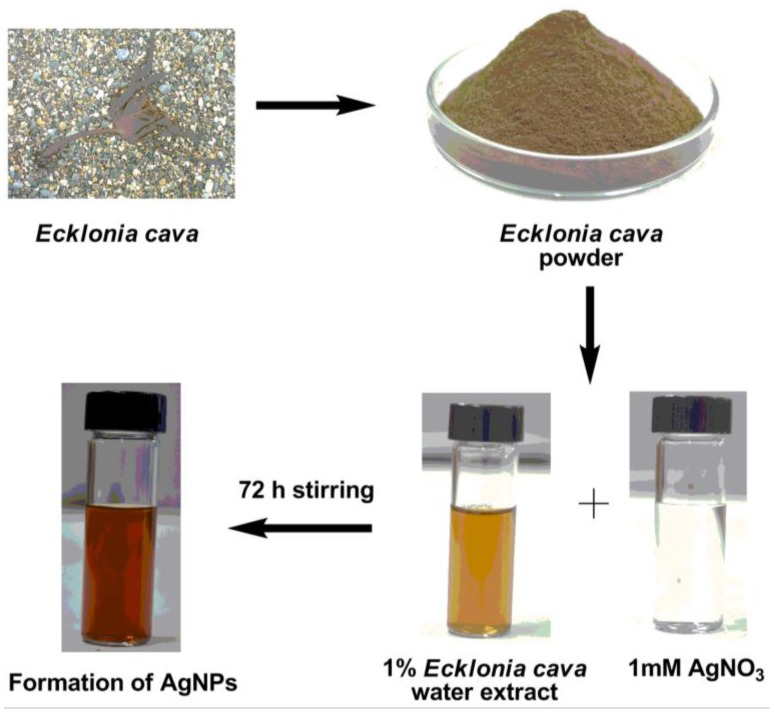
Schematic representation of green synthesis of AgNPs. *Ecklonia cava* is collected from the sea and then ground into a fine powder. The aqueous extract of *Ecklonia cava* is mixed with 1 mM AgNO_3_ solution and stirred for 72 h to synthesize AgNPs.

Aqueous extract of *Ecklonia cava* (1% *w*/*w*) was mixed with 1 mM of silver nitrate (AgNO_3_) solution at room temperature. The formation of AgNPs was confirmed by the appearance of dark yellow color, which is a typical color of AgNPs in solution due to their surface plasmon resonance (SPR) [72]. The color of reaction solution was yellowish brown upon 1 h of reaction, but it changed to dark brown color when full reduction of silver ions was completed at 72 h (Figure 2). 

**Figure 3:**
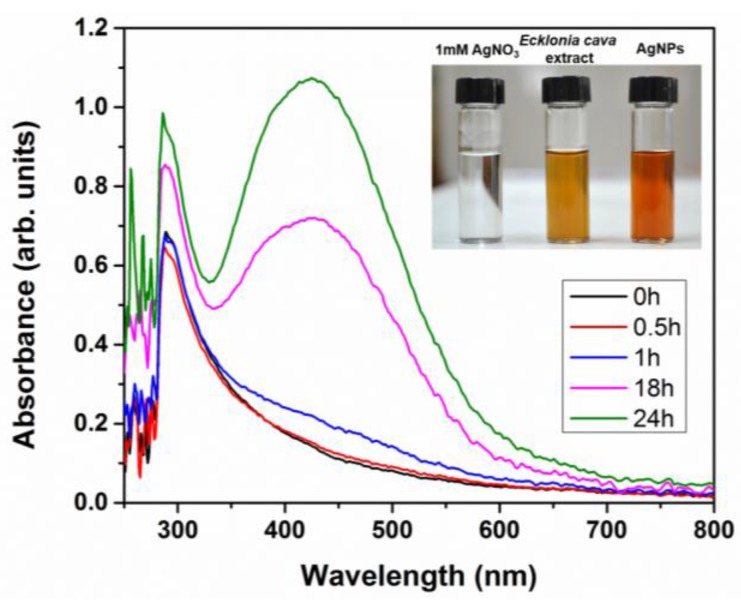
UV-Vis absorption spectra of biosynthesized AgNPs at different time intervals.

UV-Vis spectroscopy was used to confirm the synthesis of AgNPs with aqueous extract of *Ecklonia cava*. UV-Vis spectra scanned after time intervals of 0.5 h, 1 h, 18 h, and 24 h from the initiation of reaction are represented in Figure 3. Strong SPR peak of AgNPs at 418 nm was clearly observed upon 18 h of reaction, indicating the formation of AgNPs. It was also found that intensity of the SPR peak increased with reaction time (Figure 3), demonstrating the increased concentration of AgNPs. The UV-Vis spectra and visual observation demonstrate that formation of AgNPs was almost completed within 24 h. We also investigated the effect of temperature on the formation of AgNPs. When the reaction temperature increased to 200 °C, the formation of AgNPs was accelerated and thus completed within 5 h (data not shown).

### 2.2. Thermogravimetric Analysis (TGA)

**Figure 4:**
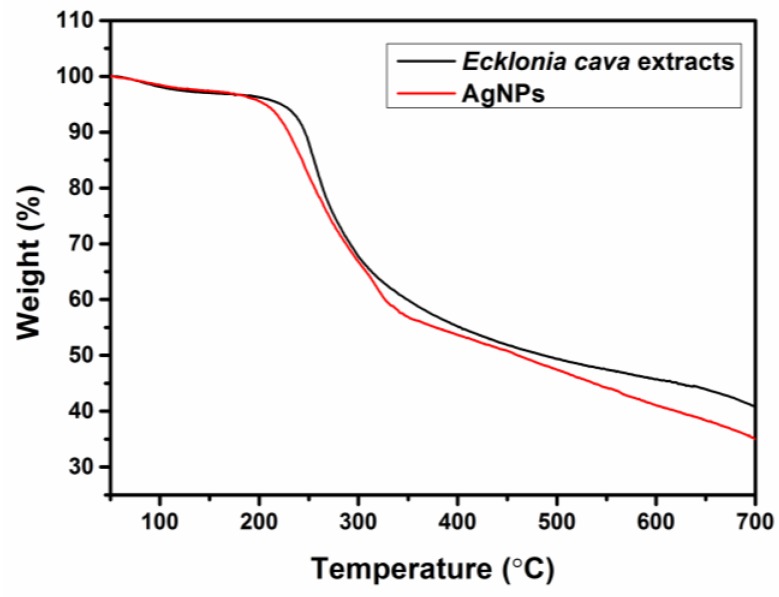
Thermogravimetric analysis of aqueous extract of *Ecklonia cava* (black curve) and biosynthesized AgNPs (red curve).

Thermal properties of *Ecklonia cava* extracts and biosynthesized AgNPs were confirmed by thermogravimetric analysis (TGA) using a Pyris 1 TGA analyzer (Perkin-Elmer, Waltham, MA, USA), as shown in Figure 4. The TGA result exhibits the strong deflection point at 230 °C for *Ecklonia cava* extracts, indicating their decomposition temperature. No significant difference has been observed in TGA curves between *Ecklonia cava* extracts and AgNPs. This result clearly indicates the presence of organic materials (i.e., *Ecklonia cava*) in the biosynthesized AgNPs.

### 2.3. Fourier Transform-Infrared (FT-IR) Spectroscopy

**Figure 5:**
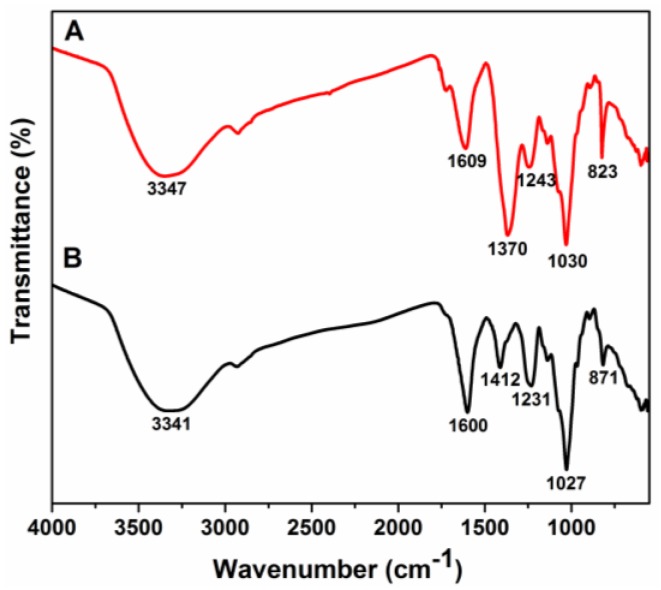
Fourier transform-infrared spectra of: (**A**) biosynthesized AgNPs; and (**B**) aqueous extract of *Ecklonia cava*.

To determine the possible biomolecules and functional groups involved in the formation of AgNPs, FT-IR spectroscopy was employed. FT-IR spectra of biosynthesized AgNPs and aqueous extract of *Ecklonia cava* were shown in Figure 5. The aqueous extract of *Ecklonia cava* showed the peaks at 871 cm^−1^, 1027 cm^−1^, 1231 cm^−1^, 1412 cm^−1^, 1600 cm^−1^, and 3341 cm^−1^. The broad peak around 3341 cm^−1^ in the spectra indicates the existence of O–H group of polyphenols or polysaccharides. The absorption band observed at 1600 cm^−1^ can be assigned to the N–H bending vibration of amine or amide groups [32]. The band observed at 1412 cm^−1^ is attributed to the C–N stretching vibration of amine or amide groups [73]. The absorption bands at 1231 cm^−1^ and 1027 cm^−1^ correspond to C–O or C–O–C stretching vibrations [74]. Similar kinds of peaks were observed at 823 cm^−1^, 1030 cm^−1^, 1243 cm^−1^, 1370 cm^−1^, 1609 cm^−1^, and 3347 cm^−1^ for biosynthesized AgNPs (Figure 5A). Similar FT-IR absorption bands from the AgNPs implies that aqueous extract of *Ecklonia cava* could act as capping agents as well as reducing agents for the formation of stable AgNPs. 

### 2.4. X-ray Diffraction (XRD) Analysis

**Figure 6:**
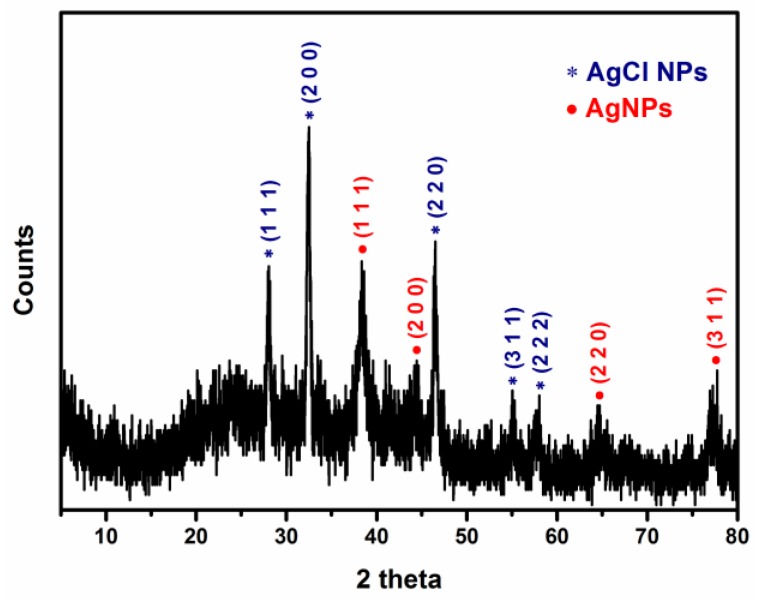
X-ray diffraction patterns of biosynthesized AgNPs (dot circle) and AgCl NPs (asterisk).

An XRD spectrum of biosynthesized AgNPs is shown in Figure 6. Distinct XRD patterns were observed at 28.0°, 32.5°, 38.2°, 44.3°, 46.4°, 55.1°, 57.5°, 64.6°, and 77.2°. The peaks at 2θ values of 38.2°, 44.3°, 64.6° and 77.2° corresponds to (1 1 1), (2 0 0), (2 2 0), and (3 1 1) planes of face-centered cubic (FCC) structure of silver, respectively (Joint Committee on Powder Diffraction Standard (JCPDS) file: 04-0783). It is in agreement with several studies that have reported similar XRD patterns of biosynthesized AgNPs [59]. It was found that other co-existing peaks at 2θ values of 28.0°, 32.5°, 46.4°, 55.1°, and 57.5° correspond to (1 1 1), (2 0 0), (2 2 0), (3 1 1), and (2 2 2) planes of face-centered cubic crystalline phase of silver chloride, respectively (JCPDS file: 31-1238) [75,76,77,78,79,80]. This result clearly indicates the production of Ag/AgCl composite nanoparticles (Ag/AgCl NPs) using aqueous extract of *Ecklonia cava*. The chloride ions might be originated from aqueous extract of *Ecklonia cava*. The formation of AgCl NPs might be attributed to the interaction of silver ions with chloride ions present in aqueous extract of *Ecklonia cava*. Similar results have been previously reported regarding the biosynthesis of AgNPs [75,78].

### 2.5. Size and Morphology Analysis of Biosynthesized AgNPs

**Figure 7:**
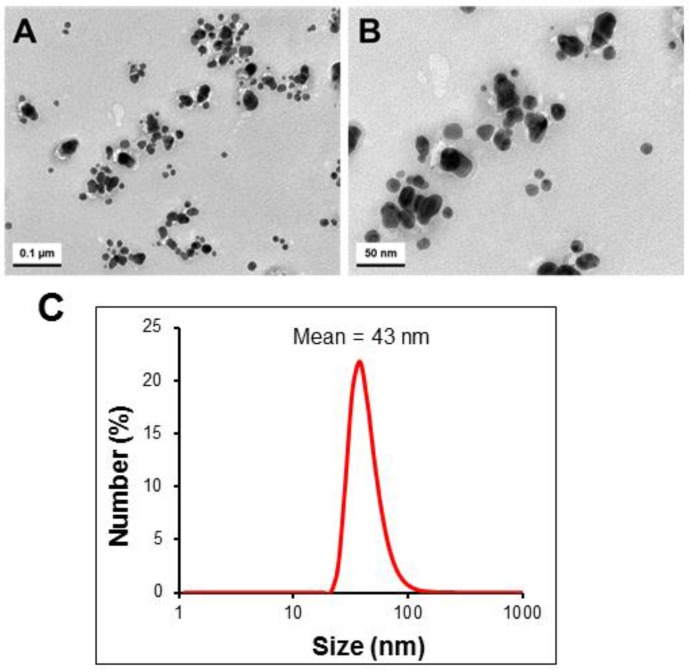
(**A**,**B**) Transmission electron microscopy images of biosynthesized AgNPs at different magnifications; and (**C**) particle size distribution of AgNPs determined by DLS.

Transmission electron microscopy (TEM) images of biosynthesized AgNPs in different magnifications were shown in Figure 7. The AgNPs were polydispersed, and their sizes were in the range of 15–30 nm (Figure 7A,B). In addition, most of them were of spherical shape. The mean hydrodynamic diameter of the AgNPs dispersed in deionized water, determined by DLS, was 43 nm with PDI of 0.27 (Figure 7C). 

### 2.6. Antimicrobial Activity by Biosynthesized AgNPs

**Figure 8:**
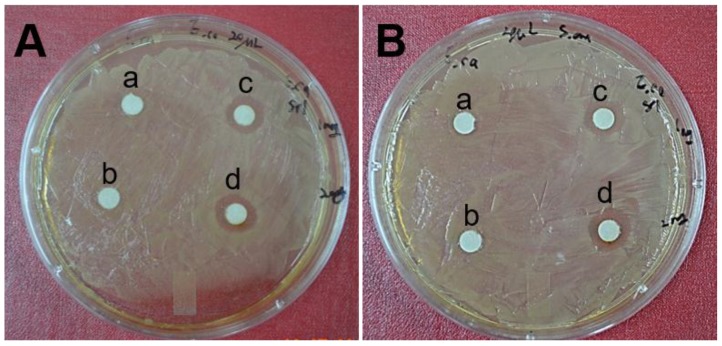
Antimicrobial activity of biosynthesized AgNPs, determined by an agar well diffusion assay. Pictures show inhibition zones produced by the biosynthesized AgNPs against *E. coli* and *S. aureus.* (**A**) *E. coli* colonies treated with: (**a**) 20 µg of aqueous extract of *Ecklonia cava*; (**b**) 40 µg of aqueous extract of *Ecklonia cava*; (**c**) 20 µg of AgNPs; and (**d**) 40 µg of AgNPs. (**B**) *S. aureus* colonies treated with: (**a**) 20 µg of aqueous extract of *Ecklonia cava*; (**b**) 40 µg of aqueous extract of *Ecklonia cava*; (**c**) 20 µg of AgNPs; and (**d**) 40 µg of AgNPs.

AgNPs were well-known to have strong antimicrobial activities [6]. Antimicrobial activity of biosynthesized AgNPs was shown in Figure 8. Antimicrobial activity of the biosynthesized AgNPs was investigated by growing *Escherichia coli* (*E. coli*) ATCC 10536 and *Staphylococcus aureus* (*S. aureus*) ATCC 6538 colonies on Luria–Bertani (LB) broth agar plates. As shown in Figure 8A, significant inhibition zone of *E. coli* was observed with the colonies treated with AgNPs, as compared to those treated with aqueous extract of *Ecklonia cava* alone. It was also clearly observed that biosynthesized AgNPs revealed a concentration-dependent antibacterial activity. *E. coli* colonies treated with 40 µg of AgNPs exhibited a larger diameter of zone of inhibition (12 ± 1 mm) (Figure 8(Ad)), as compared to those treated with 20 µg of AgNPs (10 ± 1 mm) (Figure 8(Ac)). Efficient antimicrobial activity of the biosynthesized AgNPs was also observed with *S. aureus.* The *S. aureus* colonies treated with 40 µg of AgNPs exhibited a larger diameter of zone of inhibition (Figure 8(Bd)), as compared to those treated with aqueous extract of *Ecklonia cava* alone (Figure 8(Bb)). It was reported that synthesized AgNPs with *Rhus chinensis* extracts exhibited efficient antimicrobial activity against *S. aureus, Staphylococcus saprophyticus*, *E. coli*, and *Pseudomonas aeruginosa* [61]. This study used 50 µg to 70 µg of AgNPs to achieve good antibacterial activity against all of the tested bacteria. In this study, effective antimicrobial activity was achieved by using only 40 µg of biosynthesized AgNPs, demonstrating a higher antimicrobial activity of the biosynthesized AgNPs using *Ecklonia cava* extracts. Half maximal effective concentration (EC_50_) of AgNPs against *E. coli* was found to be 15.2 µg/mL, whereas a slightly higher EC_50_ (i.e., 16.2 µg/mL) was required for *S. aureus*. 

Although AgNPs have demonstrated effective antimicrobial activities, the mechanism of action on microorganisms has not been clearly elucidated yet. It has been proposed that silver ions released from AgNPs can interact with thiol groups present in respiratory enzymes of bacterial cells, thus disrupting their respiration process [81]. Another possible mechanism of cell death is the interaction of silver ions with bases and phosphorus groups of DNA, leading to the inhibition of DNA replication and thus cell death [82].

### 2.7. Antioxidant Activity by Biosynthesized AgNPs

**Figure 9:**
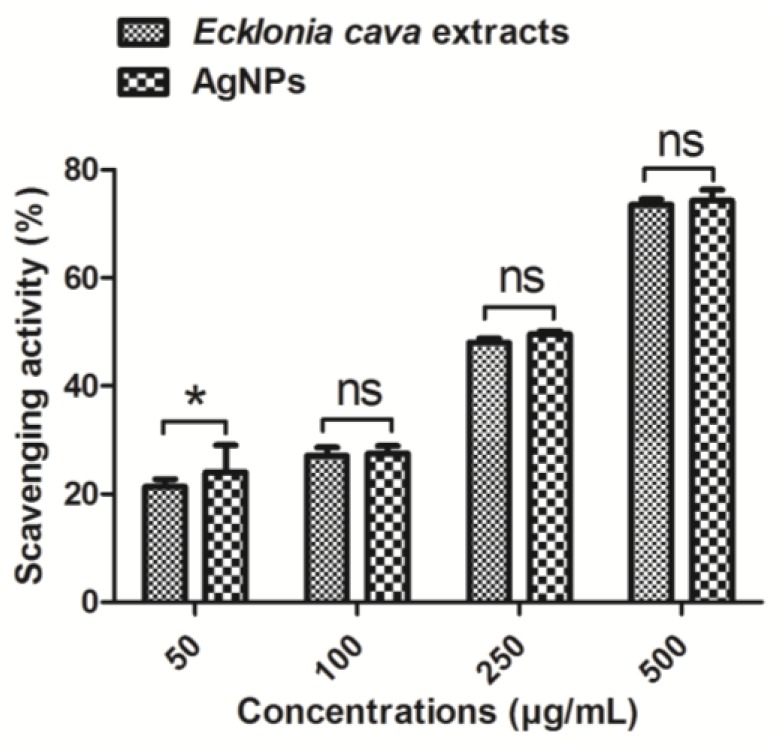
1,1-diphenyl-2-picrylhydrazyl (DPPH) radical scavenging activity of *Ecklonia cava* extract and biosynthesized AgNPs. (ns: non-significant; * *p* < 0.05).

Antioxidant activity of biosynthesized AgNPs was evaluated by 1,1-diphenyl-2-picrylhydrazyl (DPPH) radical scavenging assay (Figure 9). Free radical scavenging activity of AgNPs was determined by a decrease in absorbance of DPPH solution at 517 nm. When DPPH solution was mixed with 250 µg/mL of *Ecklonia cava* extract or biosynthesized AgNPs, ca. 50% of scavenging activity was achieved (Figure 9). DPPH radical scavenging activities of *Ecklonia cava* extract and biosynthesized AgNPs were similar at the same concentrations (e.g., 100, 250, and 500 µg/mL). High antioxidant activity of *Ecklonia cava* extract is possibly due to polyphenolic compounds, as previously reported [67]. This result indicates that strong antioxidant activity of biosynthesized AgNPs is highly related to the *Ecklonia cava* extract remained on the surface of the AgNPs. Due to the efficient antioxidant activities of both *Ecklonia cava* extracts and AgNPs, combination of AgNPs and *Ecklonia cava* with synergistic effects can be a good candidate as pharmaceutical and nutraceutical products.

### 2.8. Anticancer Activity by Biosynthesized AgNPs

**Figure 10:**
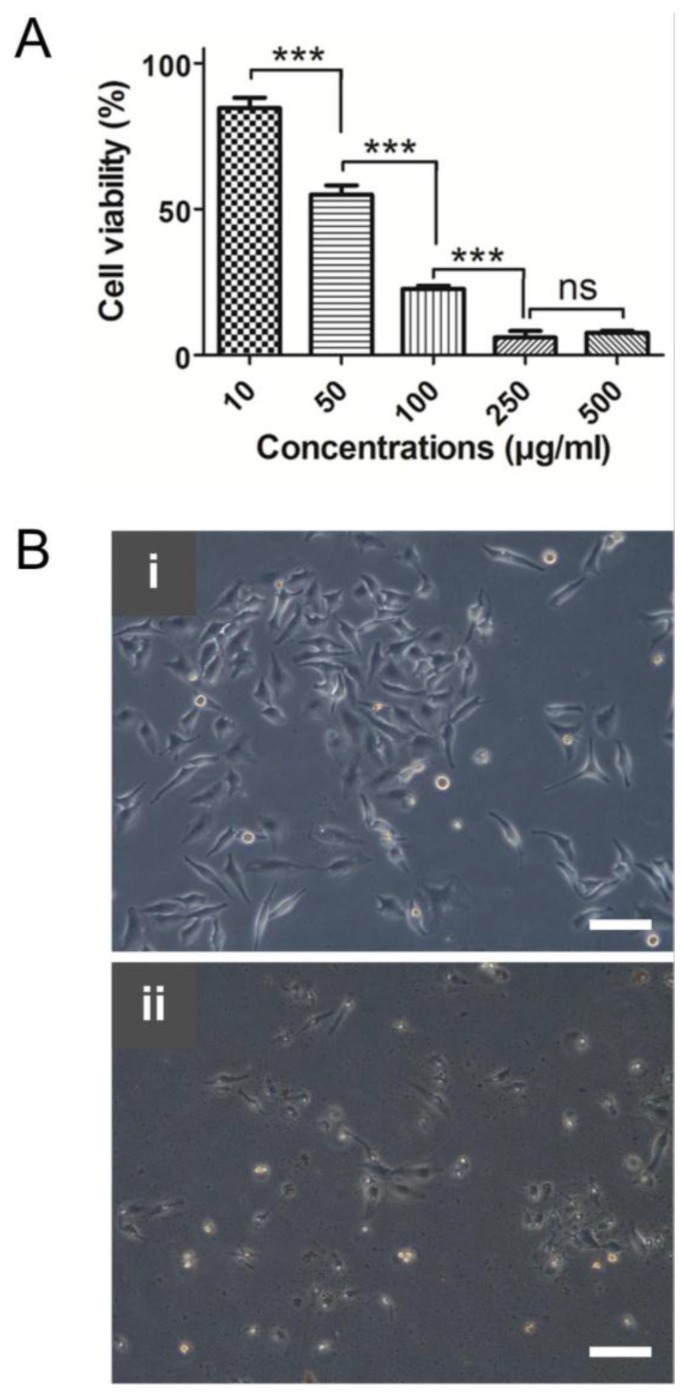
(**A**) Anticancer activity of biosynthesized AgNPs against HeLa cells (ns: non-significant; *** *p* < 0.001); and (**B**) optical microscopy images of HeLa cells after treatment with: (**i**) 250 µg/mL of *Ecklonia cava* extracts; and (**ii**) AgNPs. Scale bars = 100 µm.

In recent years, searching anticancer drug candidates from marine resources is increasing due to their lower side effects [83,84]. Anticancer activity of biosynthesized AgNPs using *Ecklonia cava* extracts was investigated by using human cervical cancer cells (HeLa cells). Figure 10A shows the cytotoxicity of AgNPs at different concentrations. IC_50_ value of the AgNPs was found to be around 59 µg/mL. The cells treated with aqueous extract of *Ecklonia cava* alone did not show any noticeable cytotoxicity at high concentrations such as 250 µg/mL (data not shown). A similar anticancer capability of biosynthesized AgNPs against HeLa cells was found in other recent studies [85]. Biosynthesized AgNPs using *Podophyllum hexandrum* exhibited an efficient anticancer activity against HeLa cells with an IC_50_ value of 20 µg/mL. High cytotoxic effect of biosynthesized AgNPs using *Cymodocea serrulata* was also reported [86]. Their IC_50_ value against HeLa cells was 34.5 µg/mL. 

Morphologies of HeLa cells after treatment with *Ecklonia cava* extracts and biosynthesized AgNPs at 250 µg/mL of concentration were observed. The cells treated with *Ecklonia cava* extracts did not show any dramatic morphological changes, whereas AgNPs led to significant morphological changes, attributed to the rupture of the membrane (Figure 10B). 

### 2.9. Apoptosis Assay

**Figure 11:**
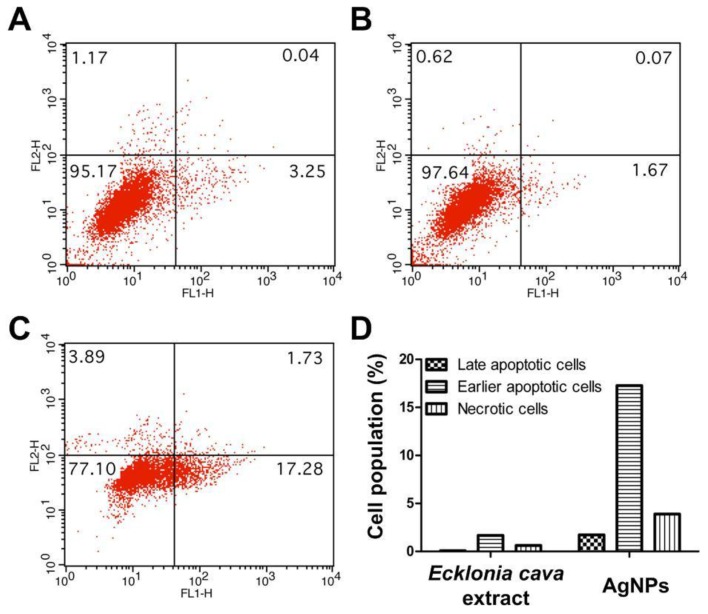
Annexin/PI staining of: (**A**) untreated HeLa cells; (**B**) HeLa cells treated with 250 µg/mL of *Ecklonia cava* extracts; (**C**) HeLa cells treated with 250 µg/mL of biosynthesized AgNPs; and (**D**) relative cell population of HeLa cells after treatment with *Ecklonia cava* extracts and biosynthesized AgNPs.

Apoptosis of HeLa cells treated with biosynthesized AgNPs at 250 µg/mL concentration was investigated using Annexin V-FITC/PI staining. The result of live cells (Annexin V^−^, PI^−^), necrotic cells (Annexin V^−^, PI^+^), early apoptotic cells (Annexin V^+^, PI^−^), and late apoptotic/dead cells (Annexin V^+^, PI^+^) is represented in Figure 11. As shown in Figure 11A,B, both untreated cells and *Ecklonia cava extracts*-treated cells remained almost viable, indicated by Annexin V^−^ and PI^−^ staining. Negligible amount of the cells underwent apoptosis. In contrast, a significant increase in the population of early apoptotic cells was detected when the cells were treated with biosynthesized AgNPs at 250 µg/mL concentration, as indicated by positive staining for Annexin V and negative staining for PI (17.28% in the lower right quadrant) (Figure 11C,D). In addition, there was an increase in the population of necrotic cells (1.73% in the upper right quadrant) for the cells treated with biosynthesized AgNPs (Figure 11C,D). This result indicates that anticancer activity of the biosynthesized AgNPs against HeLa cells are closely associated with their apoptosis induction. Similar results were reported in a previous study. AgNPs synthesized with *Moringa oleifera* showed a high anticancer activity against HeLa cells in a dose-dependent manner [8]. A significantly increased amount of the cells was identified as early apoptotic cells after treatment with the AgNPs, confirming the direct anticancer effect of apoptosis induced by the AgNPs.

## 3. Materials and Methods

### 3.1. Materials

*Ecklonia cava* powder was obtained from Jeju Island, Korea. Further fined powders after grinding were used in this study. Silver nitrate (AgNO_3_) and 1,1-diphenyl-2-picryl-hydrazyl (DPPH), [3-(4,5-dimethylthiazol-2-yl)-2,5-diphenyltetrazolium bromide] (MTT) were purchased from Sigma Aldrich (St. Louis, MO, USA). *Escherichia coli* ATCC 10536 and *Staphylococcus aureus* ATCC 6538 were purchased from Korean culture of center of microorganisms, South Korea. Luria–Bertani broth (USB Corporation, Cleveland, OH, USA) and agar (LAB M Limited, Bury, UK) were used. HeLa cells were purchased from ATCC (Manassas, VA, USA). Dulbecco’s modified eagle medium (DMEM) and fetal bovine serum (FBS) were obtained from Lonza Chemicals. Other chemicals were used in this experiment are analytical grades.

### 3.2. Preparation of Aqueous Extract of Ecklonia cava and Biosynthesis of AgNPs

Five grams of *Ecklonia cava* powder were mixed with 500 mL of deionized water at 100 °C for 1 h. The homogeneous solution was then centrifuged at 3000 rpm for 20 min, and the large particles were settled down at the bottom of the conical tube. The clear brown color solution was decanted and filtered through a filter paper. The filtrate was then stored at 4 °C until the next use. Ten milliliters of aqueous extract of *Ecklonia cava* was taken and mixed with 90 mL of 1 mM AgNO_3_ solution. A color change from yellow to dark brown indicates the formation of AgNPs by reducing Ag^+^ to Ag^0^. The dark brown color mixture solution was stirred for 72 h. The biosynthesized AgNPs were lyophilized and stored until to the next use.

### 3.3. UV-Visible Spectroscopy

Reduction of silver ions by aqueous extract of *Ecklonia cava* was monitored using UV-visible spectroscopy. An aliquot of the reaction mixture was collected periodically and scanned using a spectrophotometer (GeneQuant 1300, GE Healthcare, Piscataway, NJ, USA) at wavelengths between 200 and 800 nm with a resolution of 1 nm. 

### 3.4. Thermogravimetric Analysis

A Perkin-Elmer model of TGA-7 thermogravimetric system with a microprocessor driven temperature control unit and a TA data station was used. The mass of the samples was generally in the range of 2–3 mg. The sample pan was placed in the balance system equipment, and the temperature was raised from 50 to 700 °C at a heating rate of 10 °C per minute under nitrogen with a flow rate of 50 cm^3^/min. The mass of the sample pan was continuously recorded as a function of temperature.

### 3.5. Fourier Transform-Infrared Spectroscopy

Functional groups and chemical compositions of AgNPs were analyzed using a FT-IR spectrometer (Nicolet iS10, Thermo Electron Scientific Instruments LLC, Madison, WI, USA). FT-IR analysis of the dried AgNPs was performed in the attenuated total reflectance (ATR) mode, and the spectra were obtained in the range of 4000–400 cm^−1^. 

### 3.6. X-ray Diffraction Analysis

XRD measurement was performed on a Philips X’Pert-MPD diffractometer (Philips, Almelo, The Netherlands) with Cu Kα radiation (λ = 1.540 Å) at 30 mA and 40 kV. The scan was performed in the 2θ range from 5° to 80° at the scanning rate of 2°/min.

### 3.7. Transmission Electron Microscopy (TEM) and Dynamic Light Scattering (DLS) Analysis

Size and surface morphology of biosynthesized AgNPs were measured using a transmission electron microscope (TEM, H7500, Hitachi Ltd., Tokyo, Japan) at 120 kV. TEM grids were prepared by placing 5 μL of the AgNPs solution on carbon-coated copper grids and dried. Size distribution of the AgNPs was characterized by dynamic light scattering using a Malvern Zetasizer Nano ZS (Worcestershire, UK). 

### 3.8. Agar Well Diffusion Assay

Antimicrobial activity of biosynthesized AgNPs against microorganisms was assessed by an agar well diffusion method as previously described [61]. Briefly, both *Escherichia coli* and *Staphylococcus aureus* were cultured in 3 mL of Luria–Bertani broth. Bacteria concentration was determined by optical densities at 600 nm. Around 0.2 × 10^8^ CFU of bacteria in culture broth were plated on petri dishes. We prepared the stock (1 mg/mL) of the lyophilized *Ecklonia cava* and AgNPs. Then four paper disks containing 20 or 40 µL (equivalent to 20 µg and 40 µg) of synthesized AgNPs were placed carefully on the microbial plate. The paper disk containing aqueous extract of *Ecklonia cava* only was also used as a control group. The bacteria on the petri dishes were cultured for 24 h at 37 °C, and the antimicrobial activity of the AgNPs was measured by inhibition zones.

An MTT assay was used to determine antibacterial activities of *Ecklonia cava* extracts and AgNPs. Five hundred microliters of diluted bacteria culture (1 × 10^6^ CFU/mL) was treated with different concentrations (0, 10, 20, 30, 40 and 50 µg/mL) of *Ecklonia cava* extracts and AgNPs and kept in a shaking incubator at 37 °C for 24 h. Subsequently, 50 µL of MTT solution (5 mg/mL) was added into the samples to form formazan crystas within live bacteria. Then, the samples were centrifuged at 8000 rpm for 10 min to separate the formazan crystals. The supernatant was eliminated, and 1000 µL of dimethyl sulfoxide (DMSO) was added to dissolve the formazan crystals. Antibacterial activities were determined by measuring the absorbance of the formazan solution at 540 nm.

### 3.9. DPPH Radical Scavenging Assay

Free radical scavenging activity of *Ecklonia cava* extracts and biosynthesized AgNPs were measured using a conventional DPPH radical scavenging assay [87]. Briefly, 0.1 mM DPPH solution in ethanol was prepared, and different concentrations of *Ecklonia cava* extracts and AgNPs (e.g., 50, 100, 250, and 500 µg/mL) were mixed with DPPH solution to attain the final respective concentration. DPPH solution without sample was used as a blank. The mixture solution was vortex-mixed and then incubated for 30 min at 37 °C. Then the mixture solution was centrifuged at 3000 rpm for 5 min, and the absorbance of the supernatant liquid was measured at 517 nm using a microplate reader (GENios^®^, Tecan Austria GmbH, Grödig, Austria). DPPH scavenging activity (%) was calculated using the following formula:

$$\mathrm{DPPH}\;\mathrm{scavenging}\;\mathrm{activity}\;\left(\%\right)=\frac{\left(\mathrm{Absorbance}\;\mathrm{of}\;\mathrm{blank}\;\mathrm{sample}\;-\;\mathrm{Absorbance}\;\mathrm{of}\;\mathrm{treated}\;\mathrm{sample}\right)}{\mathrm{Absorbance}\;\mathrm{of}\;\mathrm{blank}\;\mathrm{sample}}\;\times \;100$$

### 3.10. Cytotoxicity Assay

HeLa cells were cultured with DMEM media supplemented with 10% FBS, penicillin (100 IU/mL), and streptomycin (100 µg/mL). The cells were initially seeded into a 24-well plate at the density of 5 × 10^4^ per well. After 24 h of incubation at 37 °C, the cells were treated with different concentrations of AgNPs (0–500 µg/mL). After further incubation for 24 h at 37 °C, cytotoxicity effect of AgNPs on the cells was measured using an MTT assay. Briefly, the cells were incubated with 1 mL of MTT solution (1 mg/mL) for 4 h. Then, 1 mL of dimethyl sulfoxide was added to solubilize the formed MTT formazan. Cell viability was determined by measuring the absorbance of the formazan products at 540 nm using a microplate reader. Measurements were performed in triplicates, and the concentration of AgNPs that can induce 50% of cytotoxicity was determined graphically. 

### 3.11. Optical Microscopy Analysis

HeLa cells (2 × 10^5^ cells/well) were cultured in six-well plates. After 24 h of incubation, cells were treated with aqueous extract of *Ecklonia cava* (250 µg/mL) and AgNPs, respectively. After 6 h of incubation, morphologies of the cells were observed using an optical microscope (CTR 6000, Leica, Wetzlar, Germany). 

### 3.12. Annexin V-FITC/Propidium Iodide (PI) Staining

HeLa cells were seeded into six-well plates at 3 × 10^5^ cells per well and cultured for 24 h at 37 °C. The cells were then treated with aqueous extract of *Ecklonia cava* and AgNPs at 250 µg/mL concentrations, followed by incubation for 4 h. After the cells were trypsinized, they were resuspended in 100 µL of Annexin V binding buffer and then stained with Annexin V and PI solution according to the manufacturer’s protocol (BD Biosciences, Heidelberg, Germany). The mean fluorescence intensities from the stained cells were measured using a flow cytometer (FACS Calibur, BD Biosciences). The results were expressed as a percentage of live cells (Annexin V^−^, PI^−^) necrotic cells (Annexin V^−^, PI^+^), early apoptotic cells (Annexin V^+^, PI^−^), and late apoptotic/dead cells (Annexin V^+^, PI^+^). The percentage of apoptotic cells after treatment with AgNPs was compared to that of untreated cells.

### 3.13. Statistical Analysis

All the experiments were performed in triplicates. Data were analyzed using one-way analysis of variance (ANOVA) on the significance level of *p* < 0.01 and presented as mean ± standard deviation.

## 4. Conclusions

AgNPs were successfully synthesized using marine algae *Ecklonia cava* via a simple and eco-friendly green approach for the first time. The formation of AgNPs was confirmed by UV-Vis spectroscopy. The biosynthesized AgNPs were spherical and crystalline, with an average size of 43 nm. The biosynthesized AgNPs exhibited an efficient antibacterial efficacy in a dose-dependent manner. They also showed an efficient antioxidant activity with an IC_50_ value of 198 µg/mL for DPPH. Furthermore, they greatly induced apoptosis and led to the consequent anticancer effect against human cervical cancer cells. This study demonstrates that eco-friendly and simple green synthesis of AgNPs using *Ecklonia cava* extracts could be a competitive alternative to conventional chemical methods. It is also suggested that the biosynthesized AgNPs with efficient antimicrobial, antioxidant, and anticancer activities hold huge potential for pharmaceutical, nutraceutical, and cosmeceutical applications. 

## Figures and Tables

**Table 1:** Biosynthesized AgNPs using various microorganisms.

No.	Species	Reaction Time	Size	Applications	Ref.
1	*Trichoderma viride*	48 h	5–40 nm	Antimicrobial	[13]
2	*Chaetoceros calcitrans*	2 weeks	N.A.	Antimicrobial	[22]
3	*Aspergillus clavatus*	N.A.	10–25 nm	Antifungal	[23]
4	*Porphyra vietnamensis*	15 min	13 ± 3 nm	Antibacterial	[24]
5	*Gelidiella acerosa*	48 h	22 nm	Antifungal	[25]
6	*Sargassum tenerrimum*	20 min	20 nm	Anti-bacterial	[26]
7	*Ulva fasciata*	N.A	28–41 nm	Antimicrobial	[27]
8	*Padina tetrastromatica*	24 h	14 nm	Antibacterial	[28]
9	*Turbinaria conoides*	N.A	96 nm	Antibacterial	[29]
10	*Padina pavonica*	24 h	46.8 nm	Antimicrobial	[30]
11	*Cryphonectria*	24 h	30–70nm	Antimicrobial	[31]
12	*Pterocladia capillacae*, *Jania rubins*, *Ulva faciata*, *Colpmenia sinusa*	3 h	20 nm	Antimicrobial	[32]
13	*Gracilaria corticata*	20 min	18–46 nm	Antifungal	[33]
14	*Sargassum cinereum*	3 h	45–76 nm	Antimicrobial	[34]
15	*Padina gymnospora*	N.A.	25–40 nm	Antimicrobial	[35]
16	*Sargassum plagiophyllum*	24 h	21–48 nm	Antimicrobial	[36]
17	*Sargassum longifolium*	64 h	N.A.	Antifungal	[37]
18	*Pithophora oedogonia*	N.A.	34.03 nm	Antibacterial	[38]
19	*Sesbania grandiflora*	15 min	12 nm	Antimicrobial	[39]
20	*Stenotrophomonas maltophilia*	N.A.	93 nm	Antimicrobial	[40]
21	*Croton bonplandianum*	N.A.	32 nm	Antimicrobial and anticancer	[41]
22	*Chenopodium murale*	N.A.	30–50 nm	Antimicrobial	[42]
23	*Alternanthera sessilis*	N.A.	10–30 nm	Anticancer	[43]
24	*Pseudomonas putida*	20 min	6–16 nm	Antibacterial and anticancer	[44]
25	*Citrullus colocynthis*	24 h	31 nm	Anticancer	[45]
26	*Chlorella vulgaris*	24 h	7 nm	Anticancer and antimicrobial	[46]
27	*Lovoa trichilioïdes*	2 h	37–43 nm	Anti-bacterial	[47]
28	*Ficus elastica*	30 min	50–60 nm	Air pollution control	[48]
*29*	*Ficus religiosa*	10 min	5–35 nm	In vivo antitumor	[49]
30	*Morinda pubescens*	N.A.	N.A.	Antioxidant and anticancer	[50]
31	*Saccharomyces boulardii*	4 h	3–10 nm	Anticancer	[51]
32	*Alternanthera sessilis* (Linn.)	N.A.	20–30 nm	Antimicrobial and antioxidant	[52]
33	*Citrus reticulata juice*	N.A.	N.A.	Antimicrobial	[53]
34	*Caulerpa racemosa*	24 h	5–25 nm	Antibacterial	[54]
35	*Spirogyra varians*	N.A.	17.6 nm	Antibacterial	[55]
36	*Enteromorpha flexuos*	N.A.	2–32 nm	Antimicrobial	[56]
37	*Cyanobacteria and Microalgae*	72 h	13–31 nm	Antibacterial	[57]
38	*Dracocephalum moldavica*	1 h	31 ± 6 nm	Antimicrobial	[58]
39	*Dimocarpus longan* Lour.	5 h	9–32 nm	Anticancer	[59]
40	*Curculigo orchioides rhizome*	N.A.	15–18 nm	Larvicidal and Anticancer	[60]
41	*Rhus chinensis*	12 h	150 nm.	Antibacterial	[61]

N.A. = Not Available.
